# Taking advantage of unexpected WebCONSORT results

**DOI:** 10.1186/s12916-016-0758-4

**Published:** 2016-12-05

**Authors:** Erik Cobo, José Antonio González

**Affiliations:** Statistics and Operations Research Department, Barcelona-Tech, UPC, c/ Jordi Girona 1, C5-213, 08034 Barcelona, Spain

**Keywords:** Clinical trials, CONSORT, Peer review, Research, Reporting guidelines, EQUATOR

## Abstract

To estimate treatment effects, trials are initiated by randomising patients to the interventions under study and finish by comparing patient evolution. In order to improve the trial report, the CONSORT statement provides authors and peer reviewers with a guide of the essential items that would allow research replication. Additionally, WebCONSORT aims to facilitate author reporting by providing the items from the different CONSORT extensions that are relevant to the trial being reported. WebCONSORT has been estimated to improve the proportion of reported items by 0.04 (95% CI, –0.02 to 0.10), interpreted as “no important difference”, in accordance with the scheduled desired scenario of a 0.15 effect size improvement. However, in a non-scheduled analysis, it was found that, despite clear instructions, around a third of manuscripts selected for trials by the editorial staff were not actually randomised trials. We argue that surprises benefit science, and that further research should be conducted in order to improve the performance of editorial staff.

Please see related research: http://bmcmedicine.biomedcentral.com/articles/10.1186/s12916-016-0736-x

## Background

Research should welcome and capitalise on surprises. A classic example of this occurred when Barry Marshall and Robin Warren found *Helicobacter pylori* in a person with chronic gastritis and gastric ulcers, conditions that were previously attributed to stress or spicy food [1]. When serendipity helped Alexander Fleming discover penicillin, his achievement was not in staining a lab plate, but in reproducing the results and advancing the possibilities.

There is a continuous research path from the discovery to the implementation of new interventions. At the beginning, researchers such as Fleming have ‘an idea’. At the end, they measure the intervention effect. In other words, they ask themselves: What would the evolution be for some patients if, instead of the standard intervention, we provide them the updated one. Since researchers are unable to observe both evolutions in the same patient, a fairly simple (at least, conceptually) process is chosen: randomly allocate both treatments to patients and compare their evolution. This, and nothing else, is a randomised clinical trial.

Unfortunately, things can go wrong – protocol deviations, unmasked evaluation and sample attrition have been proven to bias the results [2], and poor methodological background [3] and conflict of interests [4] may further misguide authors. Therefore, in order to facilitate reproducibility, the CONSORT statement [5] has been developed to guide them in the reporting of essential items. However, as trials with different objectives, methodologies or types of interventions may need different essential items, new extensions have been added and gathered into the EQUATOR website [6]. To facilitate manuscript preparation, WebCONSORT provides all the items that apply to the reported trial. In order to estimate the effect of WebCONSORT on manuscript completeness, Hopewell et al. [7] conducted a randomised trial in which experimental units were themselves manuscripts of randomised clinical trials, and reported that WebCONSORT improves the proportion of reported items by 0.04, although random allocation makes this result compatible (95% CI) with any true value between a decrease of 0.02 and an improvement of 0.10. The authors interpret those values as “*no important difference*” [7], in accordance with the scheduled scenario of a 0.15 effect size improvement.

### Unexpected findings – editors under the magnifying glass

Looking at additional results, Hopewell et al. [7] showed no surprise when they found “*that in a quarter (23%) of manuscripts, authors either selected an inappropriate CONSORT extension or failed to select the right extension applicable to their trial*”. As this agreed with their prior thoughts, it reinforced their aim to enhance authors’ reporting of randomised trials. However, if there is no surprise, one may feel that this is a boring result.

Nevertheless, Hopewell et al. [7] “*did not anticipate that journals would enroll manuscripts that were not in fact reports of randomised trials*”. In the Discussion, they state that: “*More than one third (39%) of registered manuscripts were excluded from the analysis as they were not reports of randomised trials. This was despite clear instructions provided to journal editorial staff, and included in the revision letter to authors, that only manuscripts reporting the results of randomised trials were eligible for inclusion. Clearly, the editorial staff at some journals were unable to correctly identify a randomised trial based on what was reported in the submitted manuscript*”. Further, in their Results section, they describe the recruitment process: “*Between 25 March 2013 and 22 September 2015, 357 manuscripts were registered on the WebCONSORT study site from 46 general medical and specialty journals with an impact factor ranging from 11.34 to 0.65 as of 2014.* […] *The percentage of eligible manuscripts varied considerably across journals (median 73%; IQR 27% to 100%).*” Thus, randomised trials constitute the methodological gold standard for assessing treatment effects, but the editorial staff of medical journals were unable to correctly classify their own manuscripts as randomised trials. Aha!

In their Discussion, Hopewell et al. [7] state: “*Better education is needed* […] *for both authors and journal editorial staff*”. This is of no surprise with regards to the authors; however, in terms of the editorial staff, it initially seems surprising, yet further consideration reveals that it may not be so. In order to assess what goes wrong along the research pipeline, the clinical scientific community, led by *JAMA* and *BMJ*, organises the quadrennial Congress on Peer Review in Biomedical Publication, which is devoted to improving the quality of biomedical literature [8, 9]. In its inception, the congress initially concentrated mainly on the peer review process – hence its name. However, the meeting now includes “*featured research describing poor practices on the parts of authors, reviewers, editors, and journals*” [8]. Thus, as editors and journals are already under the magnifying glass of investigation, this is of no surprise at all.

Unfortunately, this unanticipated, poor selection made it impossible to perform the designed intervention on misclassified manuscripts. Therefore, the WebCONSORT authors had to leave out a substantial proportion of papers, thereby losing the scheduled study power and thus making interpretation difficult. Nevertheless, the non-significance of their results should at least be interpreted carefully [10].

### Surprising unexpected results: what can we learn?

When anyone agrees to participate in clinical research – whether they be patients, recruiters, interventionists or raters – they are generously volunteering their time, efforts and data. At every stage of the pipeline, transparency implies that the scientific community has to be able to assess what went wrong. Inevitably, researchers must be unpleasant to these volunteers by asking important questions, including, Have patients followed the recommendations? Have recruiters adequately explained to them how important it is to adhere to the agreed protocol? Have interventionists applied it carefully? Have monitors made any effort to complete patient follow-up? Indeed, clinical science may never have progressed without the noble-mindedness of volunteers.

Along similar lines, while keeping in mind the generosity of everyone involved in peer review research, we should ponder the questions that the unexpected results of WebCONSORT have provided: Is there any characteristic among editors or journals that predicts the proportion of correctly classified manuscripts? For example, is impact factor involved? What is the manuscript process flow? What are the responsibilities shared by different staff members? What was their selection process? Are they professional or academic editors? What scientific and methodological background do they have? Are different scientific communities represented in their editorial staff? The answers will, ideally, lead to more ambitious research questions about the assumed causal factors: Would the modification of any of them result in the desired improvement. Obviously, this process would imply further generosity from researchers, editors and journals.

## Conclusions

The WebCONSORT randomised trial was designed to test an intervention for improving the transparent reporting of methods and results by authors. The main analysis of this objective was performed by following the most exigent experimental conditions, with the exception of the already mentioned unexpected loss of power. However, they also observed that the journal gatekeepers classified too many manuscripts as randomised trials when they were not, in fact, so. Shocking? Perhaps. Nevertheless, this post-hoc data is certainly informative and deserves further study by either the authors of the WebCONSORT study or the very same editors.

Christopher Columbus was looking for a new path to Asia. However, to the world’s surprise, he came upon America. Then, to complete the path from discovery to innovation, Columbus travelled several other times. Now, the scientific community must unravel how to improve editors’ performance.
